# Mechanical power normalized to predicted body weight is associated with mortality in critically ill patients: a cohort study

**DOI:** 10.1186/s12871-021-01497-1

**Published:** 2021-11-10

**Authors:** Yanhong Zhu, Wenyong Peng, Shuai Zhen, Xiaofeng Jiang

**Affiliations:** 1 Department of Anesthesiology, The First People’s Hospital of Pinghu, Zhejiang, China; 2 Department of Anesthesiology, Jinhua Municipal Central Hospital, 365 Renmin East Road, Jinhua, Zhejiang, China

**Keywords:** Critically ill, Mortality, Mechanical ventilation, Ventilator-induced lung injury, Mechanical power normalized to predicted body weight

## Abstract

**Background:**

Mechanical power (MP), defined as the amount of energy produced by mechanical ventilation and released into the respiratory system, was reportedly a determining factor in the pathogenesis of ventilator-induced lung injury. However, previous studies suggest that the effects of MP were proportional to their involvement in the total lung function size. Therefore, MP normalized to the predicted body weight (norMP) should outperform the absolute MP value. The objective of this research is to determine the connection between norMP and mortality in critically ill patients who have been on invasive ventilation for at least 48 h.

**Methods:**

This is a study of data stored in the databases of the MIMIC–III, which contains data of critically ill patients for over 50,000. The study involved critically ill patients who had been on invasive ventilation for at least 48 h. norMP was the relevant exposure. The major endpoint was ICU mortality, the secondary endpoints were 30-day, 90-day mortality; ICU length of stay, the number of ventilator-free days at day 28.

**Result:**

The study involved a total of 1301 critically ill patients. This study revealed that norMP was correlated with ICU mortality [OR per quartile increase 1.33 (95% CI 1.16–1.52), *p* <  0.001]. Similarly, norMP was correlated with ventilator-free days at day 28, ICU length of stay. In the subgroup analysis, high norMP was associated with ICU mortality whether low or high Vt (OR 1.31, 95% CI 1.09–1.57, *p* = 0.004; OR 1.32, 95% CI 1.08–1.62, *p* = 0.008, respectively). But high norMP was associated with ICU mortality only in low PIP (OR 1.18, 95% CI 1.01–1.38, *p* = 0.034).

**Conclusion:**

Our findings indicate that higher norMP is independently linked with elevated ICU mortality and various other clinical findings in critically ill patients with a minimum of 48 h of invasive ventilation.

**Supplementary Information:**

The online version contains supplementary material available at 10.1186/s12871-021-01497-1.

## Background

When performing surgery or in critically ill patients, mechanical ventilation is a vital component of supportive treatment since it preserves respiratory function and minimizes respiratory effort [1–3]. However, in mechanical ventilation, the mechanical force produced by the interaction between the ventilator and respiratory tract, can damage the lungs. This is known as ventilator-induced lung injury (VILI) [4–6].

The severity of VILI is determined by the ventilator settings [7]. Variables, such as tidal volume (Vt), respiratory rate (RR), and positive end-expiratory pressure (PEEP), are set directly on the ventilator by the clinician [1]. Others, such as peak pressure (PIP) and plateau pressure, rely on the patient’s respiratory system or their interaction with the ventilator. Up to now, all these factors have been assessed separately [8]; however, VILI may be unified into a single variable as mechanical power (MP), which is energy per unit of time applied to the respiratory system by the ventilator [7, 9, 10]. Energy generated by the mechanical ventilator is related to Vt, RR, PEEP, plateau pressure, and flow [9], demonstrating that MP can be calculated accurately through combining Vt, plateau pressure, PEEP, and RR [7]. The introduction of this “power equation” shows MP has a stronger modulating effect on VILI than individual ventilator settings owing to the incorporation of multiple aspects of mechanical ventilation [7, 9].

Increases in MP measured on the second day after intensive care unit (ICU) admission was correlated with increased hospital mortality in a recent study on 8207 critically ill mechanically ventilated patients [11]. In addition, previous studies [7, 8] have suggested that the effects of MP were proportional to their involvement in the total lung function size. We hypothesized that the effect of MP relative to lung size can be calculated by MP normalized to the predicted body weight (norMP) [12]. This is similar to normalizing tidal volume to predicted body weight (PBW) [13]. Therefore, norMP should outperform the absolute MP value. In acute respiratory distress (ARDS) patients, Coppola et al. [14] showed that elevated norMP led to increased mortality. However, few studies have determined the association between norMP and the outcomes of critically ill mechanically ventilated patients.

The objective of this study was to explore the prognostic role of mechanical power normalized to the predicted body weight in the clinical outcomes of intensive care patients.

## Methods

### Data source

The Massachusetts Institute of Technology’s Laboratory for Computational Physiology maintains the Multiparameter Intelligent Monitoring in Intensive Care III (MIMIC III, V.1.4) database, which includes data on over 50,000 patients admitted to the intensive care unit at Beth Israel Deaconess Medical Center between 2001 and 2012 [15]. We attended a training course on ‘protecting human subjects’ in order to apply for access to the database.

The establishment of the database was approved by the institutional review boards of the Massachusetts Institute of Technology (Cambridge, MA) and Beth Israel Deaconess Medical Center (Boston, MA). The author Jiang extracted the data for this study after passing the National Institutes of Health’s online training course (certification number: 9322422).

### Population selection criteria

In total, 58,976 intensive care unit (ICU) patients were recorded in the MIMICIII database, of these, we included in our study patients who were older than 16 at the time of their initial admission and who underwent invasive ventilation for a minimum of 48 consecutive hours. Patients were excluded if they met the criteria: had incomplete ventilatory variables to calculate MP and norMP, received pressure support ventilation, had > 1% missing data, were extubated, or had died during the first 48 h. We used only data from the patient’s initial ICU admission or initial hospitalization.

### Data extraction

The structured query language (SQL) was used to extract data from the database, and included tidal volume (Vt), positive end–expiratory pressure (PEEP), peak inspiratory pressure (PIP), RR, and the inspired fraction of oxygen (FiO_2_). The following equation was used to calculate mechanical power [7, 11]:

MP(J/min) = 0.098 × Vt × RR × (PIP – ΔP × 0.5), where the driving pressure (ΔP) = PIP – PEEP [16].

norMP (× 10^− 3^ J/min/kg) = MP/PBW [12], where PBW was the predicted body weight calculated by using the equation as used in previous studies of ventilation [17]:

$$\mathrm{PBW}=50.0+0.91\left(\mathrm{height}\ \left[\mathrm{cm}\right]-152.4\right)\ \mathrm{in}\ \mathrm{males},$$

$$\mathrm{PBW}=45.5+0.91\left(\mathrm{height}\ \left[\mathrm{cm}\right]-152.4\right)\ \mathrm{in}\ \mathrm{females}.$$

Due to the fact that the patients provided multiple measurements, the mean values obtained during the second 24 h was used. The norMP in the second day of ventilation was chosen because during the first 24 h usually mechanical ventilation is subjected to several changes and may result in more noise. Moreover, a previous study has shown that there was a decrease in MP from the first to the second 24 h of ventilation [11].

The following demographic data (using first 24 h of admission data) were collected: age, gender, ethnicity (white, black, or other), height, weight, comorbidities, and disease severity scores (Acute Physiology and Chronic Health Evaluation [APACHE] III) [18, 19]. Vital signs and laboratory measurements were captured as mean values in the first day of ventilation.

### Clinical outcome

To gather information about ICU patients’ status, the follow-up followed from ICU admission and ended at death. The major endpoint was ICU mortality, the secondary endpoints included 30-day, 90-day mortality; ICU length of stay (ICU_LOS), the number of ventilator-free days at day 28 (VFD_28, specified as the days from effective weaning to day 28; patients who died prior to weaning were considered to have no ventilator-free days).

### Statistical analyses

Continuous variables are presented in the tables as the median with interquartile ranges. The required Mann-Whitney U test, or Kruskal– Wallis test, was applied. Chisquared test or Fisher’s exact test was used for categorical variables, which are presented as a percentage. Patients were categorized into groups according to ICU mortality.

The median and interquartile range of norMP was used to classify all patients. For all outcomes, univariate and multivariate regression were used to account for potential confounding variables. Relevant covariates known to predict outcome were entered into the model including age, sex, ethnicity, BMI, admission type, comorbidities, APACHE, heart rate, MAP, SpO_2_, temperature, pH, PaO_2_ / FiO_2_, PaCO_2_. These variables were selected due to their clinical relevance. The final models were built using a stepwise backward elimination method with a significance level of 0.05. Additionally, subgroup analyses were conducted to determine the relationship between norMP and the primary outcome according to the Vt and PIP levels. According to the concepts of protective ventilation [20] and a previous study [21], and the data was empirically adjusted to define low Vt as Vt < 8 mL/PBW and low PIP as PIP < 30 cmH_2_O.

Statistical significance was described as a two-sided *p* <  0.05. SPSS software was used for all statistical analysis (SPSS-22.0; IBM Corp., Armonk, NY, USA).

## Results

**Fig. 1:**
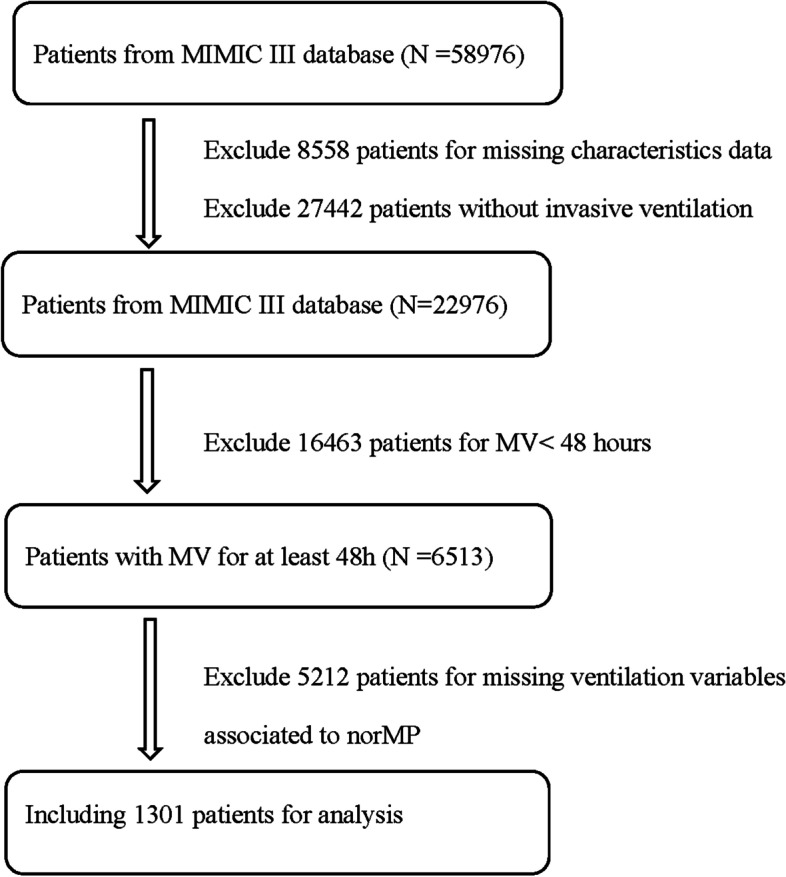
Data selection and exclusion process

Finally, 1301 patients fulfilled the requirements for the study (Fig. 1). Table 1 summarizes the demographic characteristics of survivors and non-survivors. norMP was significantly lower for survivors (222.1(161.3–288.0) × 10^− 3^ J/min/kg) than non-survivors (245.4(183.8–333.4) × 10^− 3^ J/min/kg) (*p* <  0.001), but the MP has no significant difference across the entire cohort.

**Table 1:** Comparisons of demographics between survivors and non–survivors

Baseline characteristics	Survivors (*n* = 936)	Non-survivors (*n* = 365)	*p* value
Age, years	61.3 (48.5–74.0)	68.3 (56.0–79.0)	< 0.001
Male gender	508 / 936 (54.3)	191 / 365 (52.3)	0.527
Weight, kg	82.7 (69.0–99.0)	78.0 (65.0–92.0)	< 0.001
Height, cm	170 (163–178)	168 (160–176)	0.015
BMI, kg/m^2^	28.4 (24.3–33.9)	26.8 (23.2–32.1)	0.001
PBW, kg	63.9 (54.7–73.1)	61.7 (52.5–71.8)	0.026
Admission type			0.001
Selective	82 / 936 (8.8)	11 / 365 (3.0)	
Emergency	836 / 936 (89.3)	349 / 365 (95.6)	
Urgent	18 / 936 (1.9)	5 / 365 (1.4)	
Ethnicity			0.005
White	647 / 936 (69.1)	223 / 365 (61.1)	
Black	85 / 936 (9.1)	31 / 365 (8.5)	
Other	204 / 936 (21.8)	111 / 365 (30.4)	
Comorbidities			
CHF	170 / 936 (18.1)	78 / 365 (21.4)	0.186
Cardiac arrhythmias	212 / 936 (22.6)	110 / 365 (30.1)	0.005
Valvular disease	53 / 936 (5.7)	30 / 365 (8.2)	0.090
Hypertension	143 / 936 (15.3)	56 / 365 (15.3)	0.977
Diabetes	260 / 936 (27.8)	119 / 365 (32.6)	0.085
Neurological condition	139 / 936 (14.9)	46 / 365 (12.6)	0.297
Chronic pulmonary condition	215 / 936 (23.0)	88 / 365 (24.1)	0.662
Renal failure	160 / 936 (17.1)	75 / 365 (20.5)	0.146
Liver condition	95 / 936 (10.1)	50 / 365 (13.7)	0.068
Severity of illness			
APACHE III	51 (38–69)	65 (50–85)	< 0.001
Vital signs in the beginning of ventilation			
Heart rate, bpm	89 (77–103)	89 (75–103)	0.496
MAP, mmHg	82 (76–91)	83 (76–91)	0.465
SpO_2_, %	98 (96–99)	98 (96–99)	0.016
Temperature, °C	37.0 (36.4–37.5)	36.7 (36.1–37.3)	< 0.001
Laboratory in the beginning of ventilation			
pH	7.36 (7.28–7.42)	7.34 (7.26–7.41)	0.002
PaO_2_ / FiO_2_, mmHg	238 (152–351)	213 (132–334)	0.047
PaCO_2_, mmHg	41 (37–49)	40 (34–49)	0.059
Second day of ventilation parameters			
Tidal volume, ml/kg PBW	7.8 (6.8–8.8)	7.9 (6.8–8.8)	0.880
PEEP, cmH_2_O	7 (5–10)	7 (5–10)	0.016
PIP, cmH_2_O	24 (20–28)	26 (22–30)	< 0.001
Respiratory rate, bpm	20 (17–23)	21 (18–24)	< 0.001
Minute ventilation, L/min	9.2 (7.7–11.1)	9.6 (8.6–11.7)	0.009
MP, J/min	13.5 (10.2–18.4)	14.7 (11.3–19.9)	< 0.001
norMP, 10^− 3^ J/min/kg	222.1 (161.3–288.0)	245.4 (183.8–333.4)	< 0.001

Data are median (interquartile range) or No / Total (%)

*BMI* body mass index, *PBW* predicted body weight, *CHF* congestive heart failure, *bpm* beats per minute, *SpO*_*2*_ pulse oximetry, *MAP* mean arterial blood pressure, *FiO*_*2*_ inspired fraction of oxygen, *PEEP* positive end-expiratory pressure, *PIP* peak inspiratory pressure, *MP* mechanical power, *norMP* mechanical power normalized to predicted body weight

Across the entire cohort, norMP had a median of 226.5 × 10^− 3^ J/min/kg and an interquartile range of 166.5–301.0 × 10^− 3^ J/min/kg, respectively. All patients were divided into quartile according to their norMP as follows: less than 166.4 × 10^− 3^ J/min/kg, quartile 1, (*n* = 325); from 166.5 × 10^− 3^ J/min/kg to 226.4 × 10^− 3^ J/min/kg, quartile 2 (n = 325); from 226.5 × 10^− 3^ J/min/kg to 300.9 × 10^− 3^ J/min/kg, quartile 3, (n = 325); greater than 301.0 × 10^− 3^ J/min/kg, quartile 4 (*n* = 326). The clinical outcomes of patients in various groups were summarized in Table 2. ICU mortality (72 [22.2], 91 [28.0], 85 [26.2], 117 [35.9], respectively), ICU length of stay (ICU_LOS: 7.7 [4.9–11.5], 8.1 [5.4–12.4], 9.7 [6.1–14.6], 9.8 [6.0–16.8], respectively), and ventilator-free days at day 28 (VFD_28: 21.5 [0–24.7], 21.1 [0–24.4], 19.6 [0–23.4], 15.6 [0–21.8], respectively) showed statistically significant difference (*p* <  0.05, all). However, there was no evidence that 30-day mortality and 90-day mortality between the groups was statistically different (*p* > 0.05).

**Table 2:** Clinical outcomes of subjects by the quartile of the norMP

	norMP, 10^− 3^ J/min/kg				*p* value
	quartile 1 < 166.4	quartile 2 166.4–226.4	quartile 3 226.5–300.9	quartile 4 ≥301.0	
ICU mortality	72 (22.2)	91 (28.0)	85 (26.2)	117 (35.9)	0.001
30-day mortality	102 (31.4)	105 (32.3)	104 (32.0)	123 (37.7)	0.284
90-day mortality	119 (36.6)	122 (37.5)	123 (37.8)	138 (42.3)	0.442
ICU_LOS, day	7.7 (4.9–11.5)	8.1 (5.4–12.4)	9.7 (6.1–14.6)	9.8 (6.0–16.8)	< 0.001
VFD_28, day	21.5 (0–24.7)	21.1 (0–24.4)	19.6 (0–23.4)	15.6 (0–21.8)	< 0.001

Data are median (interquartile range) or No / Total (%)

*norMP* mechanical power normalized to predicted body weight, *ICU* intensive care unit, *LOS* length of stay, *VFD_28* Ventilator-free days at day 28

**Fig. 2:**
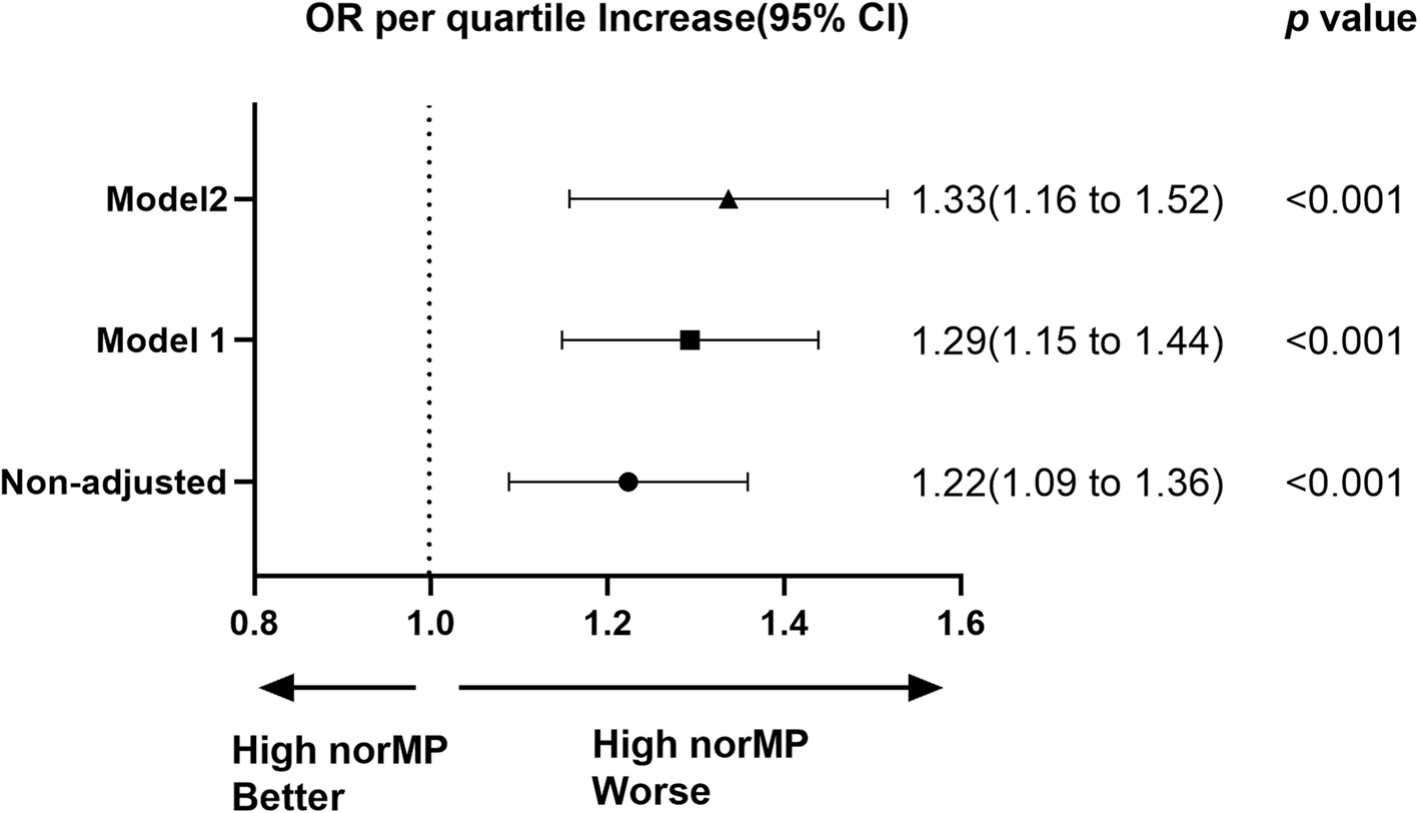
norMP in the second 24 h of ventilation and ICU mortality. Model 1 was adjusted for the confounders age, sex and ethnicity. Model 2 was adjusted for the confounders, including age, sex, ethnicity, BMI, admission type, comorbidities, APACHE, heart rate, MAP, SpO_2_, temperature, pH, PaO_2_ / FiO_2_, PaCO_2_. The odds ratio represents the odds of death per quartile increase in norMP. norMP: mechanical power normalized to predicted body weight

Figure 2 illustrates the results of the univariate and multivariate analysis of the primary outcome. Crude outcome shows that High norMP was associated with increased ICU mortality (OR = 1.22, 95% CI 1.09–1.36, *p* <  0.001). In addition, norMP in the second 24 h still had a strong correlation with increased ICU mortality even after adjustment for covariates (OR = 1.33, 95% CI 1.16–1.52, *p* <  0.001).

**Fig. 3:**
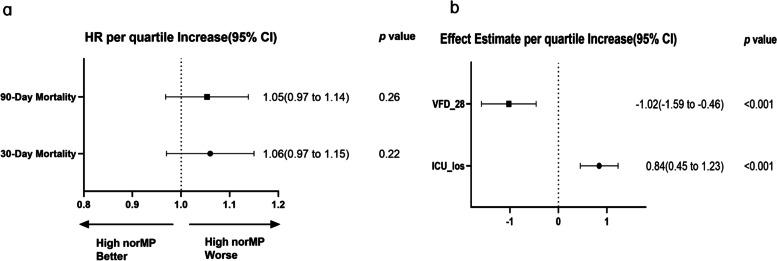
norMP in the second day of ventilation and secondary outcomes. **a** Odds ratio represents the odds of death per quartile increase in norMP. **b** Effect estimates and 95% confidence interval from the multivariable linear regression for VFD_28 and ICU_los. Effect estimate refers to the change in the outcome variable per quartile increase in norMP. norMP: mechanical power normalized to predicted body weight; VFD_28: Ventilator-free days at day 28; ICU: intensive care unit; LOS: length of stay

Figure 3 illustrates the results of the multivariate analysis of the 30-day mortality, 90-day mortality, ICU_LOS, and VFD_28. norMP in the second 24 h of ventilation was also associated with ICU length of stay and the number of ventilator-free days (Fig. 3b). However, there was no association between norMP and 30-day mortality or 90-day mortality (Fig. 3a).

**Fig. 4:**
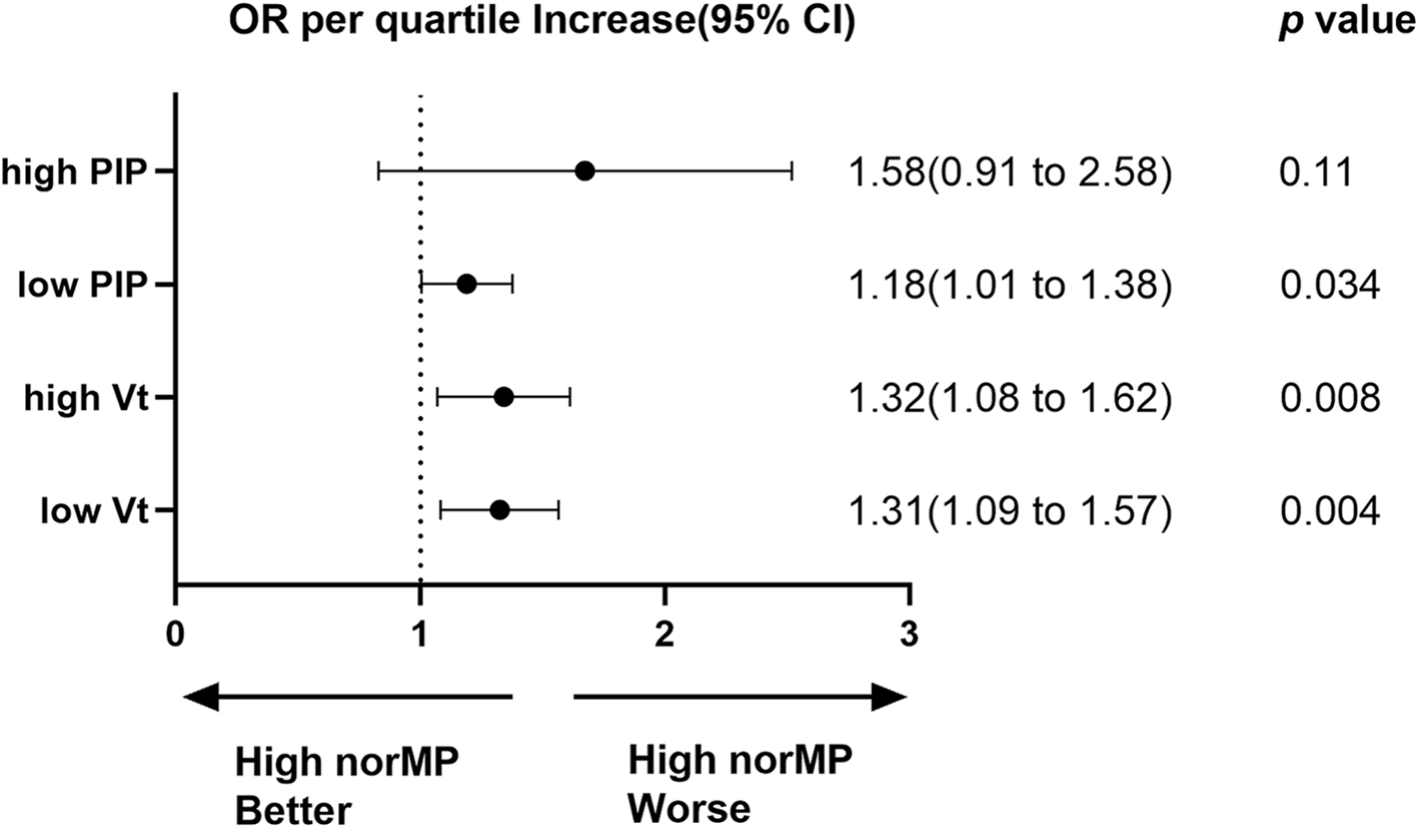
Subgroup analysis of the association between norMP and ICU mortality according to different tidal volumes and airway pressure levels. The odds ratio represents the odds of death per quartile increase in norMP. norMP: mechanical power normalized to predicted body weight; Vt: tidal volume; PIP: peak inspiratory pressure

In the subgroup analysis (Fig. 4), regardless of the Vt level, high norMP was associated with ICU mortality (OR = 1.31, 95% CI 1.09–1.57, *p* = 0.004; OR = 1.32, 95% CI 1.08–1.62, *p* = 0.008, respectively). The analysis revealed (Fig. 4) that high norMP was associated with ICU mortality only in patients with PIP < 30 cmH_2_O (OR = 1.18, 95% CI 1.01–1.38, *p* = 0.034). Our results also (eTable 1) showed the PEEP levels were significantly lower for patients with low PIP levels (5 [5–8.5]; *p* = 0.004) than for those with high PIP levels (10 [7.5–15], *p* <  0.001).

## Discussion

The essential findings of this research can be summarized as follows: (a) norMP during the second 24 h of ventilation was independently correlated with increased ICU mortality of critically ill patients, who received invasive ventilation for more than 48 h; (b) increased norMP was independently correlated with a longer ICU stay, a lower number of ventilator-free days and alive at day 28; and (c) high norMP was associated with ICU mortality regardless of Vt, but high norMP was associated with ICU mortality in patients with low PIP only.

In invasive ventilation, a lung-protection ventilation strategy that provides adequate gas exchange while minimizing VILI should be used [22, 23]. VILI has been primarily associated with excessive pressure, excessive volume, and atelectasis [8, 24]. Therefore, mechanical ventilation strategies to reduce VILI have sought to optimize all potential determinants including respiratory rate, tidal volume, and PEEP [17, 25–29]. While individual ventilator parameters have been extensively studied in previous research, few studies have considered these factors comprehensively. One study has shown that MP can be computed from its components: Vt, plateau pressure, flow, PEEP, and RR [7]. Since MP is a composite of these variables, it may be a candidate variable to improve prediction of clinical outcomes (such as mortality) [11]. The authors believe that norMP is superior to MP because the effects of MP were proportional to their involvement in total lung function size. Given similar mechanical power values, different energies will be delivered according to different ventilated lung surfaces. Hong et al. [30] have demonstrated that the effect size of MP differs across subgroups of acute respiratory failure (ARF) populations. The effect size of MP on mortality is the smallest in class 1 (baseline) and the largest in class 3 (refractory respiratory failure). The heterogeneity of patients with ARF supports the hypothesis that the effect of MP on VILI is dependent on the functional lung size. Additionally, a previous study [12], normalizing the mechanical power to the predicted body weight as a proxy for lung size, demonstrated that norMP had the highest area under the receiver operating characteristic among all ventilator parameters, and it had a more accurate prediction of in-hospital mortality. Similar to a previous study, the results of this analysis proved that norMP was a predictor of poor outcomes in ICU patients undergoing invasive ventilation.

Since the two important factors of ventilator parameters are tidal volume and airway pressure, we evaluated the effect of norMP on the prognosis of patients with different tidal volumes and airway pressure levels. In line with our hypothesis, we discovered that high MP was correlated with ICU mortality, even when Vt was low. This suggested that norMP added more information aside from the volume. Our research also demonstrated that a high norMP was associated with ICU mortality in patients with low PIP only. We propose a possible explanation for this finding. Our results showed that PEEP levels were higher in the group of patients with high PIP levels than in their counterpart. Patients with high PIP levels may be more severely ill, as sicker patients may be default be receiving higher PEEP levels. This may explain why low norMP was not associated with decreased ICU mortality in patients with high PIP. The majority of mechanical power in patients with higher levels of PEEP may be secondary to the applied PEEP. This further emphasizes the need to consider the PEEP component in the analysis. We failed to do a simple sensitivity analysis taking out the PEEP (focused on patients without PEEP), because the sample became very small in this situation. However, we have performed an analysis to determine the relationship between norMP and ICU mortality according to the different level of PEEP. Considering this analysis complicated the results so much, we decided not to report. Therefore, further investigations (including clinical trials) are necessary to explore the relationship between norMP and mortality in patients without applied PEEP.

These findings suggested that norMP might be a useful marker to predict clinical outcomes because it combines the effects of different ventilator parameters. Modifying a single parameter has little effect on the amount of energy transmitted to the lung tissue, and it does not always protect the lungs [31]. According to the concept of protective ventilation, a decrease in volume necessitates an increase in the respiratory rate to offset the loss of minute volume. A higher respiratory rate leads to higher norMP. Therefore, volume reduction does not result in profit, according to our current study and previous studies [32, 33]. In the future, ventilators directly displaying the norMP applied to the respiratory system will promote lung protection. The caregiver can titrate ventilation to reduce the amount of energy supplied to the lung tissue.

In addition to not considering the PEEP component in the analysis, our current analysis had some limitations. First, in order to determine patients with more serious illnesses and ample exposure time, only patients who underwent invasive ventilation for at least 48 h were selected. However, the current findings cannot be generalized for patients who were extubated or died within the first 48 h. Second, norMP was calculated only once and not during the ICU stay. Therefore, it did not accurately represent the temporal changes in norMP administered to the patient. Third, since the datasets used in this study were from publicly available data, the airway pressure may not have been collected under consistent standard conditions. Such is the case in patients without spontaneous breathing efforts. Finally, it was difficult to quantify functional lung size. In the present study, we indirectly described the functional lung size through PBW. However, other conditions leading to decreased functional lung size, such as ARF and ARDS, were not considered. In the future, further studies investigating normalizing MP to respiratory system compliance or lung volume determined using CT [34] are necessary in subgroup of patients with ARF or ARDS. In addition, it is hard to recognize ARF patients without lung injury and those with ventilation failure caused by neuromuscular dysfunction in MIMIC III. Thus, this issue was not analyzed in this study.

## Conclusions

High norMP is independently correlated with increased ICU mortality and many other clinical outcomes in critically ill patients who undergo invasive ventilation for at least 48 h. Due to the ease with which norMP can be determined using ventilator parameters, monitoring norMP can help predict the early outcome of ICU patients undergoing invasive ventilation.

## Supplementary Information

**Additional file 1: eTable 1**. Comparisons of PEEP between different PIP level.

## Data Availability

The datasets of the current study are available from the corresponding author on reasonable request.

## Abbreviations

BMI: Body mass index
PBW: Predicted body weight
CHF: Congestive heart failure
bpm: Beats per minute
SpO_2_: Pulse oximetry
MAP: Mean arterial blood pressure
FiO_2_: Inspired fraction of oxygen
PEEP: Positive end-expiratory pressure
PIP: Peak inspiratory pressure
RR: Respiratory rate
Vt: Tidal volume
MP: Mechanical power
norMP: Mechanical power normalized to predicted body weight
ICU: Intensive care unit
LOS: Length of stay
VFD_28: Ventilator-free days at day 28
ARF: Acute respiratory failure

## References

[1] Cruz FF, Ball L, Rocco P (2018). Ventilator-induced lung injury during controlled ventilation in patients with acute respiratory distress syndrome: less is probably better. Expert Rev Respir Med.

[2] Silva PL, Ball L, Rocco PA (2019). Power to mechanical power to minimize ventilator-induced lung injury?. Intensive Care Med Exp.

[3] Silva PL, Negrini D, Rocco PR (2015). Mechanisms of ventilator-induced lung injury in healthy lungs. Best Pract Res Clin Anaesthesiol.

[4] Beitler JR, Malhotra A, Thompson BT (2016). Ventilator-induced lung injury. Clin Chest Med.

[5] Chiumello D, Pristine G, Slutsky AS (1999). Mechanical ventilation affects local and systemic cytokines in an animal model of acute respiratory distress syndrome. Am J Respir Crit Care Med.

[6] Gattinoni L, Carlesso E, Cadringher P (2003). Physical and biological triggers of ventilator-induced lung injury and its prevention. Eur Respir J Suppl.

[7] Gattinoni L, Tonetti T, Cressoni M (2016). Ventilator-related causes of lung injury: the mechanical power. Intensive Care Med.

[8] Tonetti T, Vasques F, Rapetti F (2017). Driving pressure and mechanical power: new targets for VILI prevention. Ann Transl Med.

[9] Cressoni M, Gotti M, Chiurazzi C (2016). Mechanical Power and Development of Ventilator-induced Lung Injury. Anesthesiology.

[10] Marini JJ, Rocco PRM, Gattinoni L (2020). Static and dynamic contributors to ventilator-induced lung injury in clinical practice. Pressure, energy, and power. Am J Respir Crit Care Med.

[11] Neto AS, Deliberato RO, Johnson AEW (2018). Mechanical power of ventilation is associated with mortality in critically ill patients: an analysis of patients in two observational cohorts. Intensive Care Med.

[12] Zhang Z, Zheng B, Liu N (2019). Mechanical power normalized to predicted body weight as a predictor of mortality in patients with acute respiratory distress syndrome. Intensive Care Med.

[13] Linares-Perdomo O, East TD, Brower R (2015). Standardizing predicted body weight equations for mechanical ventilation tidal volume settings. Chest.

[14] Coppola S, Caccioppola A, Froio S (2020). Effect of mechanical power on intensive care mortality in ARDS patients. Crit Care.

[15] Johnson AE, Pollard TJ, Shen L (2016). MIMIC-III, a freely accessible critical care database. Sci Data.

[16] van Meenen DA-O, Serpa Neto A, Paulus F (2020). The predictive validity for mortality of the driving pressure and the mechanical power of ventilation. Intensive Care Med Exp.

[17] Brower RG, Matthay MA, Morris A (2000). Ventilation with lower tidal volumes as compared with traditional tidal volumes for acute lung injury and the acute respiratory distress syndrome. N Engl J Med.

[18] Salluh JI, Soares M (2014). ICU severity of illness scores: APACHE, SAPS and MPM. Curr Opin Crit Care.

[19] Zhang Z, Chen K, Chen L (2015). APACHE III outcome prediction in patients admitted to the intensive care unit with Sepsis associated acute lung injury. PLoS One.

[20] Serpa Neto A, Schultz MJ, Slutsky AS (2015). Current concepts of protective ventilation during general anaesthesia. Swiss Med Wkly.

[21] Gattinoni L, Marini JJ, Collino F (2017). The future of mechanical ventilation: lessons from the present and the past. Crit Care.

[22] Chiumello D, Brochard L, Marini JJ (2017). Respiratory support in patients with acute respiratory distress syndrome: an expert opinion. Crit Care.

[23] Coppola S, Caccioppola A, Froio S (2019). Dynamic hyperinflation and intrinsic positive end-expiratory pressure in ARDS patients. Crit Care.

[24] Albaiceta GM, Blanch L (2011). Beyond volutrauma in ARDS: the critical role of lung tissue deformation. Crit Care.

[25] Gattinoni L, Marini JJ, Pesenti A (2016). The "baby lung" became an adult. Intensive Care Med.

[26] Curley GF, Laffey JG, Zhang H (2016). Biotrauma and ventilator-induced lung injury: clinical implications. Chest.

[27] Amato MB, Meade MO, As S (2015). Driving pressure and survival in the acute respiratory distress syndrome. N Engl J Med.

[28] Putensen C, Theuerkauf N, Zinserling J (2009). Meta-analysis: ventilation strategies and outcomes of the acute respiratory distress syndrome and acute lung injury. Ann Intern Med.

[29] Meade MO, Cook DJ, Guyatt GH (2008). Ventilation strategy using low tidal volumes, recruitment maneuvers, and high positive end-expiratory pressure for acute lung injury and acute respiratory distress syndrome: a randomized controlled trial. JAMA.

[30] Hong Y, Chen L, Pan Q (2021). Individualized mechanical power-based ventilation strategy for acute respiratory failure formalized by finite mixture modeling and dynamic treatment regimen. EClinicalMedicine.

[31] Gattinoni L, Tonetti T, Quintel M (2018). Intensive care medicine in 2050: ventilator-induced lung injury. Intensive Care Med.

[32] Santos RS, Maia LA, Oliveira MV (2018). Biologic impact of mechanical power at high and low tidal volumes in experimental mild acute respiratory distress syndrome. Anesthesiology.

[33] Marini JJ, Gattinoni L (2018). Energetics and the root mechanical cause for ventilator-induced lung injury. Anesthesiology.

[34] Gattinoni L, Caironi P, Pelosi P (2001). What has computed tomography taught us about the acute respiratory distress syndrome?. Am J Respir Crit Care Med.

