# Supplementary material for: Mechanical power normalized to predicted body weight is associated with mortality in critically ill patients: a cohort study
Source: BMC Anesthesiol. 2021 Nov 10;21:278. doi: 10.1186/s12871-021-01497-1 (PMC8578006; doi:10.1186/s12871-021-01497-1)
Supplement: Supplementary file 1 — Additional file 1: eTable 1. Comparisons of PEEP between different PIP level. [file 12871_2021_1497_MOESM1_ESM.docx]

eTable 1. Comparisons of PEEP between different PIP level

|  | | PIP level | | | *p* value |
| --- | --- | --- | --- | --- | --- |
|  | Low PIP  ≤30 mmHg | | High PIP  >30 mmHg |  | |
| PEEP, mmHg | 5 (5 – 8.5) | | 10 (7.5 – 15) | < 0.001 | |

Data are median (interquartile range)

PEEP: positive end-expiratory pressure; PIP: peak pressure
